# Advancing aircraft engine RUL predictions: an interpretable integrated approach of feature engineering and aggregated feature importance

**DOI:** 10.1038/s41598-023-40315-1

**Published:** 2023-08-18

**Authors:** Yazan Alomari, Mátyás Andó, Marcia L. Baptista

**Affiliations:** 1 Faculty of Informatics, Institute of Computer Science, ELTE Eötvös Loránd University, Budapest, Hungary; 2 Faculty of Aerospace Engineering, Delft University of Technology, Delft, The Netherlands

**Keywords:** Engineering, Mechanical engineering

## Abstract

In this study, we present a comprehensive approach for predicting the remaining useful life (RUL) of aircraft engines, incorporating advanced feature engineering, dimensionality reduction, feature selection techniques, and machine learning models. The process begins with a rolling time series window, followed by the extraction of a multitude of statistical features, and the application of principal component analysis for dimensionality reduction. We utilize a variety of feature selection methods, such as Genetic Algorithm, Recursive Feature Elimination, Least Absolute Shrinkage and Selection Operator Regression, and Feature Importances from a Random Forest model. As a significant contribution, we introduce the novel aggregated feature importances with cross-validation (AFICv) technique, which ranks features based on their mean importance. We establish a selection criterion that retains features with a cumulative mean sum equal to 70%, thereby reducing the complexity of machine learning models and enhancing their generalizability. Four machine learning regression models—Natural and Extreme Gradient Boosting, Random Forest, and Multi-Layer Perceptron—were employed to evaluate the effectiveness of the selected features. The performance of our proposed method is assessed by the evaluation metrics Root Mean Square Error (RMSE) and R2 Score, and also considered within-interval percentages and relative accuracy metrics. Importantly, a novel PCA interpretability was introduced to provide real-world context and enhance the utility of our findings for domain experts. Our results indicate that the proposed AFICv technique efficiently achieves competitive performance across the Commercial Modular Aero-Propulsion System Simulation (C-MAPSS) sub-datasets using a significantly smaller subset of features, thus contributing to a more effective and interpretable RUL prediction methodology for aircraft engines.

## Introduction

Prognostics and Health Management (PHM) has emerged as a critical field in modern engineering systems, aiming to enhance the reliability, maintainability, and safety of these systems^1^. A critical aspect of PHM is predicting the Remaining Useful Life (RUL) of a system or component, which allows for effective decision-making in terms of maintenance and repair^2,3^. In recent years, time series data analysis has played a vital role in RUL estimation, as it can reveal hidden patterns and trends in the data that can be leveraged for better predictions^4,5^.

The complexity of model-based approaches for nonlinear systems has prompted increased focus on data-driven techniques, especially deep learning methods, for predicting RUL^6–8^. This shift is driven by the significant progress made in the field of artificial intelligence theories and practices. Commonly, factors and operational conditions, such as temperature, pressure and load in thermal systems, which can signify a component’s state, are sampled in time, resulting in a time series data. As time-dependent properties are crucial, various Recurrent Neural Network (RNN) models, including long-short term memory (LSTM), Bidirectional LSTM (Bi-LSTM)^9^ and deep LSTM frameworks^10,11^ are widely adopted to retain historical equipment states for evaluating current health conditions. Convolutional neural networks (CNN)^11,12^ have also been employed in RUL prediction to attain improved accuracy. In addition to neural networks, researchers are investigating statistical probability methods like particle filtering (PF) and extended Kalman filtering (EKF)^13^.

The Complex Systems Monitor for Advanced Propulsion System Simulation (C-MAPSS) dataset is a widely used benchmark in the field of RUL estimation and prognostics^14^. It consists of four sub-datasets (FD001, FD002, FD003, and FD004) multivariate time series data collected from aircraft engines, representing a challenging problem due to the complex nature of the data and the presence of multiple operating conditions and failure modes. Feature engineering and feature selection are essential steps in preprocessing the C-MAPSS dataset which is utilized in this article as well to obtain accurate RUL predictions^15^.

Feature engineering techniques, such as rolling window aggregation and extraction of time series features, have been employed to transform the original time series data into a more informative representation, suitable for machine learning algorithms^16,17^. In parallel, various feature selection methods have been explored to identify the most relevant and informative features for RUL estimation, including Genetic Algorithms (GA), Recursive Feature Elimination (RFE), LASSO, and Random Forest feature importance (FIRF)^18^.

## Literature review

The literature on RUL estimation and prognostics has grown substantially over the past few years. Several studies have focused on feature engineering^19–21^, feature selection^22–24^, and machine learning algorithms for improving RUL prediction accuracy^25–28^.

Feature engineering techniques have been widely explored in the context of RUL estimation. Heimes et al.^29^ proposed a technique to extract features from time series data using time-domain, frequency-domain, and wavelet-domain methods. Similarly, Lei et al.^30^ conducted a systematic review of machinery health prognostics, focusing on the entire process from data acquisition to RUL prediction. Their review covered various methods, including wavelet packet decomposition, artificial neural networks, and other machine learning techniques applied to different types of machinery and datasets, such as rolling bearings^31^ and the C-MAPSS dataset.

Wang et al.^32^ introduced a similarity-based prognostics approach for estimating the remaining useful life (RUL) of engineered systems. The authors proposed a method that calculates the similarity between the current health state of the system and the historical health states of other systems by using a weighted dynamic time warping algorithm. The RUL is then estimated based on the most similar historical health states. The proposed approach was validated using the C-MAPSS dataset, demonstrating its effectiveness in predicting the RUL of systems based on the similarities between their health states.

Deep learning, an emergent subfield of artificial intelligence, boasts the ability to autonomously and precisely construct a hierarchy of features, progressively synthesizing higher-level features from those at lower levels^33^. Deep-Convolution-Based LSTM Network for RUL Prediction^34^ and RUL Prediction Based on a Double-Convolutional Neural Network Architecture^12^ present innovative deep learning methodologies for RUL prediction in mechanical and industrial applications, specifically for rotating machinery and bearings. The former combines convolutional and LSTM layers to extract time and frequency domain features from vibration data, outperforming state-of-the-art methods in terms of accuracy and robustness. Meanwhile, the latter proposes a double-CNN architecture, utilizing an intermediate reliability variable and a mapping algorithm to enhance RUL prediction accuracy. Both studies underscore the promise and superiority of these deep learning models over traditional statistical and machine learning models, suggesting their broad applicability across complex systems.

Sheng et al.^35^ presented a novel self-adapting deep learning network (CSDLN) for predicting the RUL of aero-engines and wind turbines, overcoming limitations of prior methods such as inflexible feature learning patterns and low prediction accuracy. Their concise self-adapting deep learning network (CSDLN) model, integrating a multi-branch 1D involution neural network (MINN) for feature extraction and a trend recognition unit (TRU) for degradation trend identification, has demonstrated superior prediction accuracy and generalization in comparative and ablation experiments on their confidential WTGB dataset.

Sheng et al.^36^ introduced an innovative deep learning model, the Multi-cellular Long Short-Term Memory (MCLSTM), for predicting the RUL of aero-engines. This method leverages the capabilities of MCLSTM alongside a deep neural network to discern health indicators from raw data and accurately project engine degradation trends. When juxtaposed with a range of existing methodologies, including statistical models, shallow learning, and other deep learning approaches, Sheng et al.’s MCLSTM model demonstrated superior performance, signifying a notable advancement in RUL prediction techniques.

Feature selection has been another crucial area of research in RUL estimation. Several researchers have investigated the effectiveness of various feature selection techniques for RUL prediction. For example, Saeys et al.^18^ conducted a comprehensive review of feature selection techniques in bioinformatics, which has inspired the application of these methods to prognostics and health management problems. Another study by He et al.^37^ aimed to address the uncertainty in the prediction of RUL for lithium-ion batteries by employing a fusion of multiple data sources and models. The proposed method combined the strengths of Dempster–Shafer theory in handling uncertain and incomplete information with the flexibility of the Bayesian Monte Carlo method for model updating. Validation results demonstrated that their method effectively accounted for the uncertainties and provided accurate RUL predictions. Christ et al.^16^ introduced the TSFresh algorithm for extracting relevant features from time series data. Their work has been influential in the application of time series feature extraction techniques to RUL estimation tasks.

GA have also been employed in feature selection for RUL estimation. Lei et al.^38^ used a GA to select features for predicting the RUL of rolling bearings. Their work demonstrates the potential of evolutionary algorithms for feature selection in RUL estimation problems.

Some researchers have also explored the combination of feature selection and machine learning algorithms for RUL estimation. Zhang et al.^39^ proposed a method combining principal component analysis (PCA) and support vector regression for RUL estimation of aircraft engines. Wang et al.^32^ combined feature selection based on mutual information with deep learning for RUL prediction. These studies demonstrate the effectiveness of integrating feature selection and machine learning techniques for RUL estimation tasks.

In summary, the literature on RUL estimation and prognostics has explored various feature engineering, feature selection, and machine learning techniques. While significant progress has been made in this area, further research is needed to identify the optimal combination of preprocessing techniques and prediction models for RUL estimation.

In this article, the impact of various feature engineering and feature selection methods was investigated on RUL estimation performance using the C-MAPSS dataset. Specifically, the effectiveness of rolling window aggregation, TSFresh features extraction, GA, RFE, Lasso, and FIRF for selecting the most informative features for RUL prediction was explored. Furthermore, evaluating the performance of several machine learning algorithms, including neural networks, on the selected features to determine the optimal combination of preprocessing techniques and prediction models for RUL estimation. Additionally, a novel interpretation of Principal Component Analysis (PCA) loadings was introduced. This approach illuminates the intricate relationships between sensor readings, uncovering new narratives in the data that contribute to our understanding of engine behavior.

## Methodology

**Figure 1:**
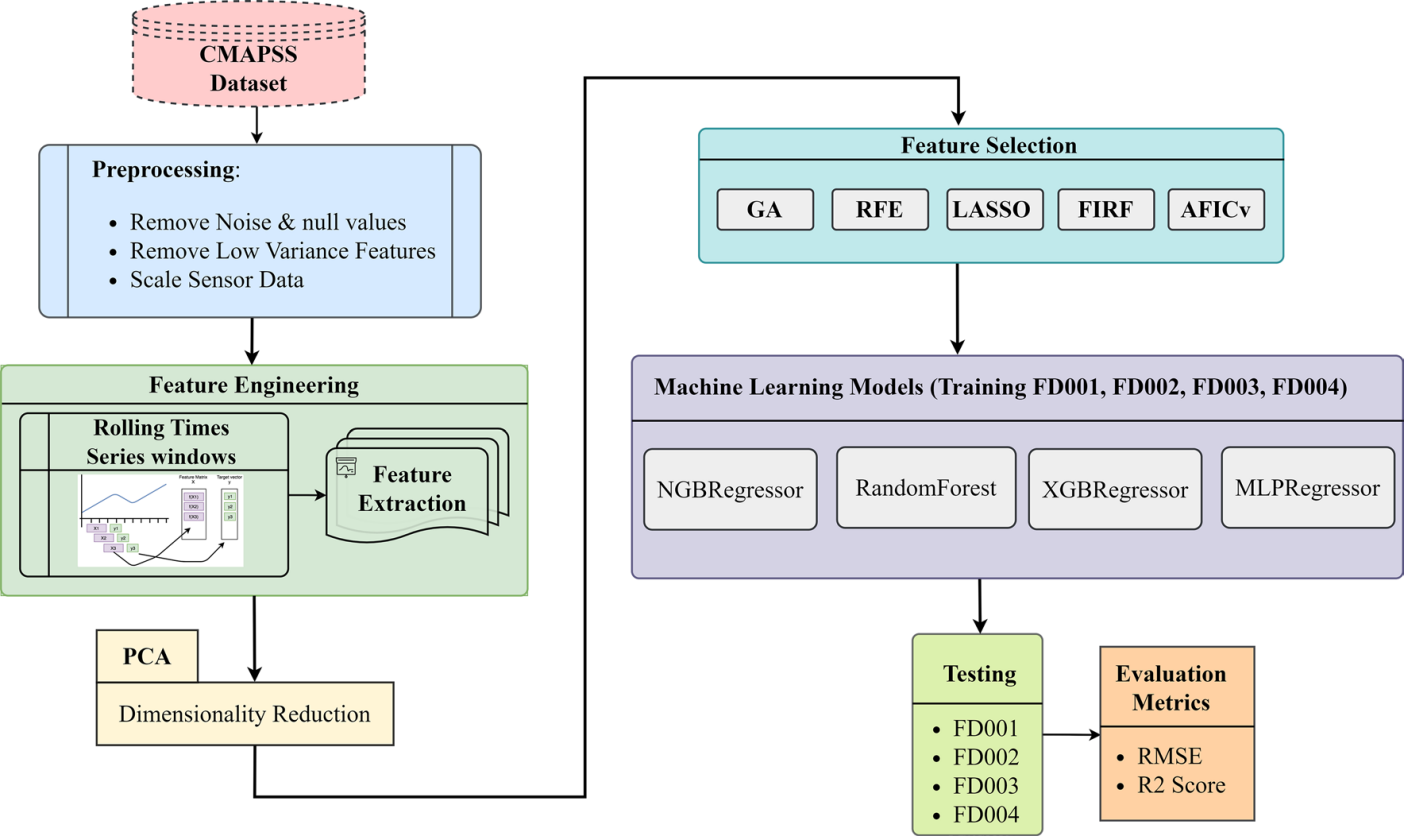
Flowchart illustrating the proposed workflow.

The methodology employed in this study, illustrated in Fig. 1, follows a structured data processing pipeline encompassing several stages. The first step is Data Preprocessing, involving the removal of low variance features and scaling of sensor data. This is followed by the Rolling Time Series Windows phase, wherein time-series features are extracted. Principal Component Analysis (PCA) is then applied to reduce the dimensionality of the feature set. Subsequently, Feature Selection is performed using five distinct techniques. This comprehensive data processing and modeling approach is finalized with a training and testing phase. The developed machine learning models are trained on a substantial portion of the dataset, and subsequently tested on unseen data. Model performance is evaluated using RMSE and the coefficient of determination (R2 score), providing a comprehensive understanding of their prediction capabilities and generalizability.

### Data preprocessing

It starts by removing features with low variance. Features with variance below a specified threshold are considered less informative for the model and are removed from the dataset. This step helps in reducing noise and computational complexity during the subsequent steps.

Next (Scaling Sensor Data) the sensor data is standardized using the standard scaler Eqs. (1)–(3) within the first n cycles of each engine, where n is a user-defined parameter. In the proposed research the value of *n* was 10, this value is based on the assumption that the first 10 cycles are representative of the engine’s normal operating condition.

The idea behind Standard Scaler is that it will transform the raw data such that its distribution will have a mean value 0 and standard deviation of 1.

1 $$Standardization\, Z= \frac{x- \mu }{\sigma },$$

2 $$Mean\,\, \mu = \frac{1}{n}{\sum }_{i=1}^{n}({x}_{i}),$$

3 $$Standard\, deviation\, \sigma = \sqrt{\frac{1}{n}{\sum }_{i=1}^{n}({{x}_{i}- \mu )}^{2}}.$$

This step is crucial to ensure that the data from different engines are on a comparable scale, which can improve the performance of subsequent steps in the pipeline. The standardization process involves subtracting the mean and dividing by the standard deviation for each sensor column within the specified range of cycles.

### Feature engineering

To capture the temporal dependencies in the sensor data, which are important for RUL prediction, rolling windows of time-series data for each engine have been generated. The rolling window process is controlled by three parameters:

- *Minimum time shift* The minimum number of time steps to shift the time series data.
- *Maximum time shift* The maximum number of time steps to shift the time series data.
- *Rolling direction* The direction of the rolling window (forward or backward).

In the proposed study the optimal values for Min_shift, max_shift, and rolling direction were 5, 20 and 1 (forward) respectively.

**Figure 2:**
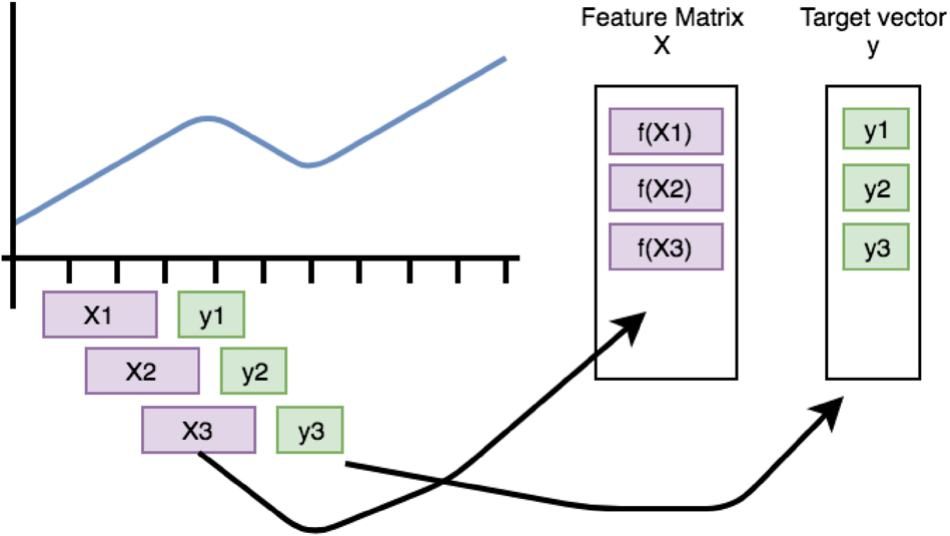
Rolling mechanism demonstration^16^.

The rolling technique is employed to convert a single time series into multiple time series, with each one terminating one (or n) time step after its predecessor. Figure 2 is an example demonstrates the overall rolling mechanism. *Tsfresh’s* rolling utilities streamline the process of reorganizing and rolling the data into an appropriate format for implementing the conventional *tsfresh.extract_features()* method. Consequently, the extraction of time series windows and the feature extraction phases are conducted independently^16^.

In this stage, the rolled time series data undergoes a comprehensive feature extraction process (Extracting Time-Series Features). A multitude of statistical features are calculated to better represent the inherent patterns and trends within the sensor data. To achieve this, a custom-defined list (as shown in Table 1) of feature extraction functions is employed. These functions perform various transformations and calculations on the data to capture unique aspects of the time series. By generating a diverse set of features, the extracted information is intended to provide a more comprehensive representation of the sensor data's underlying characteristics.

**Table 1:** Extracted time-series features.

Extracted time-series features	
Mean	First location of maximum and minimum
Mean change	Last location of maximum and minimum
Standard deviation	Time reversal asymmetry statistic^40^
Root mean square	Partial autocorrelation
Autocorrelation	Third order auto-cumulant^41^
Maximum	Complexity invariant distance—cross entropy^42^
Minimum	
Linear trend (*attributes: intercept, slope, and standard error*)	
Augmented Dickey Fuller test (*attributes: test statistic, p-value*)^43^	
Linear Trend Timewise (*attributes: intercept and slope*)	
Lempel Ziv complexity (*with bins 2, 3, 5, 10, and 100*)^44^	
Permutation entropy (*with tau 1 and dimensions 3, 4, 5, 6, and 7*)^45^	
Fast Fourier Transform Coefficient (*coefficients 0 to 10, attribute: absolute value*)^46^	
Fast Fourier Transform Aggregated (*aggregate types: centroid, variance, skew, and kurtosis*)^46^	

### Principal component analysis (PCA)

Post the feature engineering phase, principal component analysis (PCA) was utilized as a dimensionality reduction technique. PCA projects the original high-dimensional extracted features onto a lower-dimensional subspace, aiming to retain as much of the original variance as possible^47^. It’s important to note that PCA was not applied directly to the raw data, but to the high-dimensional feature space obtained from the extraction process.

The number of principal components is a user-defined parameter. In this study, we selected 15 principal components, which jointly explain approximately 50% of the variance in the extracted feature set. This decision was made based on a balance between preserving essential information captured by the features and avoiding potential overfitting that could degrade the model's performance on unseen data.

Table 2 presents the variance explained by each of the 15 principal components and the cumulative variance explained:

**Table 2:** Principal components variance and cumulative variance.

Principal component	Explained variance	Cumulative variance
1	0.210	0.210
2	0.130	0.340
3	0.051	0.391
4	0.021	0.413
5	0.015	0.428
6	0.011	0.440
7	0.008	0.449
8	0.007	0.456
9	0.006	0.463
10	0.006	0.469
11	0.006	0.475
12	0.006	0.481
13	0.005	0.487
14	0.005	0.493
15	0.005	0.499

#### Principal component loading

The output of PCA can often be challenging to interpret in the context of the original features. This is where PCA loadings come into play.

PCA loadings^47^ provide insight into how each original feature in the dataset contributes to the newly created principal components. Specifically, a loading represents the correlation between a particular original feature and a principal component, thereby informing us about the degree and direction of the influence of each original feature on each component.

In the context of multivariate data, such as multiple sensor readings from an aircraft engine, PCA loadings can help us interpret the transformed features (principal components) in terms of the original sensor readings (“Principal component loading interpretability” section). By understanding the loadings, we can extract meaningful insights about the underlying structure of our data. For example, we might discover that certain sensor readings are collectively important for characterizing specific aspects of engine performance or behavior. By examining PCA loadings carefully, we can translate the mathematical transformations of PCA back into the real-world context of our dataset.

### Feature selection

The final step in the pipeline is feature selection using five different methods like GA, RFE, LASSO Regression, FIRF, and Aggregated feature importance’s with cross validation (AFICv). Supplementary Appendix 1 contains the details of the feature selection techniques according to the literature.

The AFICv works by selecting the features that have the cumulative mean sum equal to 70% (threshold). This helps to reduce the number of selected features, and show how the prediction (RUL) can change in case of less feature.

After training, the feature importance’s from each cross-validated model was extracted and aggregated them to calculate the mean and standard deviation of importance for each feature. With this information, the features were ranked based on their mean importance in descending order.

### Dataset description

The C-MAPSS (Commercial Modular Aero-Propulsion System Simulation) dataset is a comprehensive collection of data that simulates the performance and degradation of large commercial turbofan aircraft engines. The dataset is composed of four distinct sub-datasets (FD001, FD002, FD003, and FD004), which represent data collected from 21 sensors simulating the degradation of large commercial turbofan aircraft engines, as provided by NASA^48^. This dataset documents various engine flight conditions and fault modes, with each sub-dataset containing both a training and test set. Table 3 outline the specific composition of the C-MAPSS dataset.

**Table 3:** Composition of the C-MAPSS dataset.

Dataset	FD001	FD002	FD003	FD004
No. of engines in the training set	100	260	100	249
No. of engines in the test set	100	259	100	248
Operating conditions	1	6	1	6
Fault modes	1	1	2	2
Training size	20,632	53,760	24,721	61,250
Test size	13,097	33,992	16,597	41,215

## Experimental results

### Feature selection

Feature selection serves as a critical parameter for achieving accurate predictions while minimizing computational time. The appropriateness of feature selection is contingent upon the specific dataset being utilized. In the context of the FD001 dataset, there are 15 principal components that could potentially function as features. Table 4 delineates the features chosen in accordance with various techniques, including GA, RFE, LASSO, FIRF, and AFICv.

**Table 4:** Selected features in case of FD001 dataset.

Method	No. of selected features	Selected features
GA	12	[0, 1, 2, 3, 4, 5, 6, 7, 9, 10, 12, 14]
RFE	10	[0, 2, 3, 4, 5, 6, 7, 8, 13, 14]
LASSO	10	[0, 1, 2, 3, 4, 5, 6, 7, 8, 11]
FIRF	12	[0, 2, 3, 4, 5, 6, 7, 8, 9, 11, 12, 14]
AFICv	5	[0, 1, 2, 4, 6]

The results of the feature selection techniques, such as GA, RFE, LASSO, and FIRF, has resulted in selecting 10 to 12 features for model training as presented in Table 4 for the dataset FD001. However, in AFICv technique a custom-predefined threshold (cumulative mean sum) value was assigned to 70% (“Feature selection” section) which resulted in a selection of only five features. Table 5 shows the features for the four datasets ranked based on their mean importance in descending order and highlighting the first 3–5 features that have a cumulative mean sum equal to roughly 0.7.

**Table 5:**
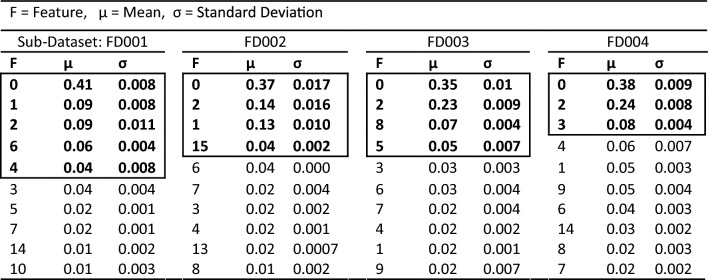
Aggregated feature importance’s with cross validation at 70% threshold.

However, by employing AFICV, a significant reduction in the number of selected features was achieved based on the selected threshold. Only 3–5 features were used to train the model. This reduction in complexity (less feature selection) not only simplifies model interpretation but also potentially enhances generalization by mitigating the risk of overfitting.

### Model evaluation

The proposed study was evaluated on the C-MAPSS dataset (FD001, FD002, FD003, and FD004) by training four different machine learning models (NGBRegressor, RandomForestRegressor, XGBRegressor, and MLPRegressor) on the extracted features from the mentioned feature selection methods. The prediction performance of the model is characterized by the error between predicted RUL and real RUL. Therefore, RMSE and coefficient of determination (R2 Score) which are defined by the Eqs. (4)–(7), were applied for performance evaluation, where *n* is the number of data points. Table 6 shows the performance of the different ML models utilizing the different feature selection techniques for the dataset FD001.

4 $$RMSE \left({P}_{RUL}, {T}_{RUL}\right)= \sqrt{\frac{1}{n}\sum_{i=1}^{n}{({P}_{RUL}- {T}_{RUL})}^{2}},$$

5 $${R}^{2}=1- \frac{sum\, of\,squares\, of \,residuals\,\left(RSS\right)}{total \,sum\, of \,squares\, \left(TSS\right)},$$

6 $$\begin{gathered} RSS = \mathop \sum \limits_{i = 1}^{n} \left( {T_{RUL} - P_{RUL} } \right)^{2} \hfill \\ P_{RUL} = Predicted \,RUL\,value \hfill \\ T_{RUL} = True\, RUL \,value, \hfill \\ \end{gathered}$$

7 $$\begin{gathered} TSS = \mathop \sum \limits_{i = 1}^{n} \left( {T_{RUL} - {\upmu }_{{T_{RUL} }} } \right)^{2} \hfill \\ {\upmu }_{{T_{RUL} }} = Mean\, value \,of \,True \,RUL. \hfill \\ \end{gathered}$$

**Table 6:** Evaluation metrics FD001.

Evaluation metric	RMSE					R2 score				
Selection method	GA	RFE	LASSO	FIRF	AFICv	GA	RFE	LASSO	FIRF	AFICv
NGBRegressor	12.6	12.5	12.6	12.6	**12.4**	0.9	0.90	0.90	0.90	**0.91**
RandomForestRegressor	12.7	12.4	12.9	12.3	**12.4**	0.9	0.91	0.90	0.91	**0.90**
XGBRegressor	12.8	12.1	11.7	12.5	**11.9**	0.9	0.91	0.91	0.90	**0.91**
MLPRegressor	12.5	12	13.3	11.9	**11.8**	0.9	0.91	0.89	0.91	**0.91**

Significant values are given in bold.

To ensure replicability and provide a basis for future investigations and improvements, we provide a comprehensive overview of the final hyperparameters and the construction of the MLPRegressor and CNN models in Table 7.

**Table 7:** MLPRegressor and CNN model configuration.

Hyperparameter/attribute	MLPRegressor values	CNN values
Number of hidden layers	5	2
Nodes in each hidden layer	(10, 20, 30, 20, 10)	Conv1D: 64, Dense: 64
Activation function	ReLU	ReLU
Solver for weight optimization	Adam	Adam
Regularization parameter (alpha)	0.001	None
Early stopping	Enabled	Enabled
Validation fraction (for early stopping)	0.2	0.2
Loss function	Squared loss	Mean squared error
Number of trained parameters (weights)	1710	20,801
Number of epochs trained	101	100
Learning rate	0.001 (default “Adam”)	0.001 (default “Adam”)
Momentum	0.9 (default “Adam”)	0.9 (default “Adam”)

The performance of the five feature selection techniques employed in this study is found to be quite similar when applied to the FD001 sub-dataset. In order to streamline the presentation of results for the other sub-datasets (FD002, FD003, and FD004), as shown in Table 8, only GA technique, which resulted in the highest number of selected features, was compared with the AFICv technique, which identified the lowest number of features. This comparison provides valuable insights into the impact of feature selection on model performance.

**Table 8:** Evaluation metrices in case of FD002–FD003–FD004 datasets using GA and AFICv.

C-MPASS sub-dataset	FD002				FD003				FD004			
Evaluation metric	RMSE		R2 score		RMSE		R2 score		RMSE		R2 score	
Feature selection method	GA	AFICv	GA	AFICv	GA	AFICv	GA	AFICv	GA	AFICv	GA	AFICv
NGBRegressor	23.7	**23.4**	0.80	**0.80**	15	**14.9**	0.86	**0.87**	23.5	**23.4**	0.80	**0.81**
RandomForestRegressor	23.7	**23.7**	0.80	**0.80**	16.1	**16.2**	0.84	**0.84**	23.6	**23.4**	0.80	**0.81**
XGBRegressor	23.9	**23.6**	0.80	**0.80**	15.3	**15.3**	0.86	**0.86**	22.5	**22.4**	0.81	**0.82**
MLPRegressor	23.1	**23**	0.81	**0.81**	15	**14.6**	0.86	**0.86**	23.7	**22.3**	0.83	**0.83**

Significant values are given in bold.

While the performance metrics in Table 8, such as RMSE and R2 score, are quite similar for both methods across the three C-MAPSS sub-datasets (FD002, FD003, and FD004), it is important to note the stark difference in the number of features utilized by each method. The GA method selected 12 features, whereas AFICv only required 3–5 features to achieve comparable or slightly improved performance.

This observation suggests that AFICv can effectively identify a smaller subset of the most important features, leading to more efficient and potentially more interpretable models without sacrificing prediction accuracy. In practical applications, the reduced number of features may lead to faster training and prediction times, as well as lower computational costs. The comparable performance between the two methods, despite the difference in the number of selected features, highlights the effectiveness of AFICv as a feature selection technique in the context of the C-MAPSS datasets and the four machine learning models considered in this study.

Table 9 presents a performance comparison of the proposed method with other approaches from the literature, revealing the effectiveness of the MLPRegressor and CNN with AFICv in predicting the RUL of engines across the C-MAPSS sub-datasets. Our selected features, when used in these models, notably improved the results, demonstrating their versatility and effectiveness across various complex deep learning layers. The proposed method outperforms Neural Network (NN)^15^, Deep Neural Network (DNN)^49^, Convolutional Neural Network (CNN)^49^, and Long Short-Term Memory Networks (LSTM)^15^ in terms of RMSE for sub-datasets FD001, FD002, and FD004. This highlights the superior predictive capability of our proposed method for these datasets.

**Table 9:** Performance comparisons of different methods on the C-MAPSS dataset characterized by RMSE.

C-MPASS sub-dataset	FD001	FD002	FD003	FD004
Neural network (NN)^15^	14.80	25.64	15.22	25.80
Deed neural network (DNN)^15^	13.56	24.61	13.93	24.31
Convolutional neural network (CNN)^49^	18.45	30.29	19.82	29.16
Long short-term memory networks (LSTM)^15^	13.52	24.42	**13.54**	24.21
BiLSTM^50^	13.65	23.18	13.74	24.86
Similarity-based^51^	16.43	23.36	17.43	23.36
**CNN with AFICv**	**11.7**	**23**	14.4	**22.8**
**Proposed MLPRegressor with AFICv**	**11.8**	**23**	14.6	**22.3**

Significant values are given in bold.

However, it is important to note that the proposed method does not yield the best results for sub-dataset FD003. In this case, the LSTM^15^ method achieves slightly better performance. This suggests that although the proposed method demonstrates strong performance overall, there may still be room for improvement in specific scenarios. Future research could explore the reasons behind this discrepancy and investigate potential enhancements to the proposed method to further improve its predictive accuracy across all C-MAPSS sub-datasets.

### Predictions

**Figure 3:**
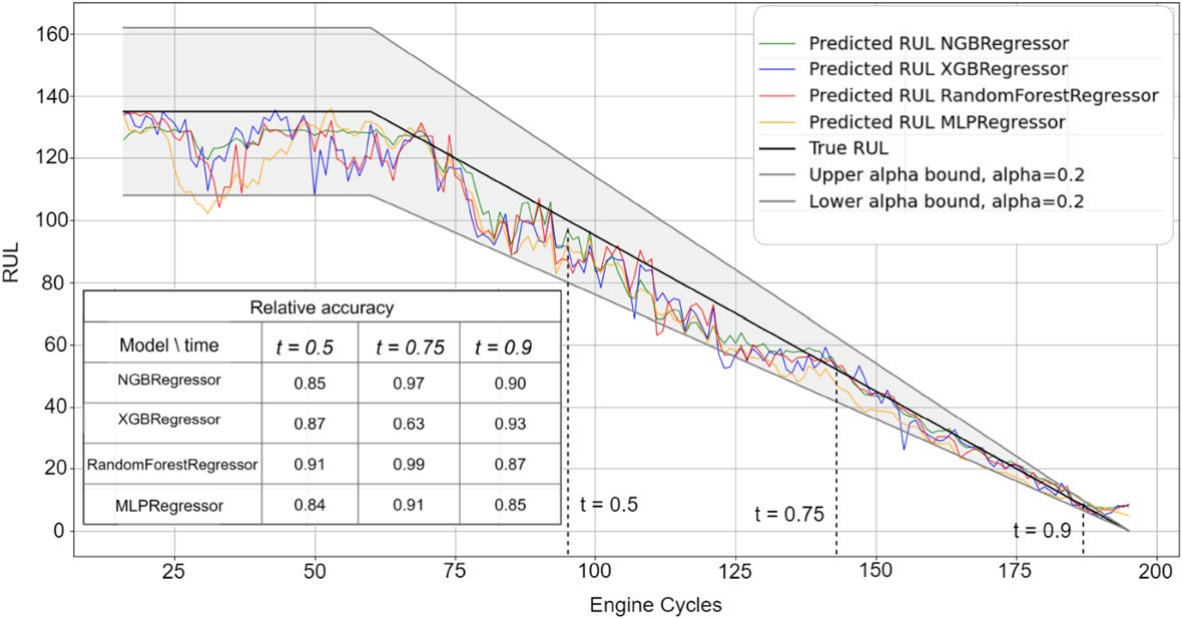
Predicted vs true RUL for engine #34 dataset FD001.

**Figure 4:**
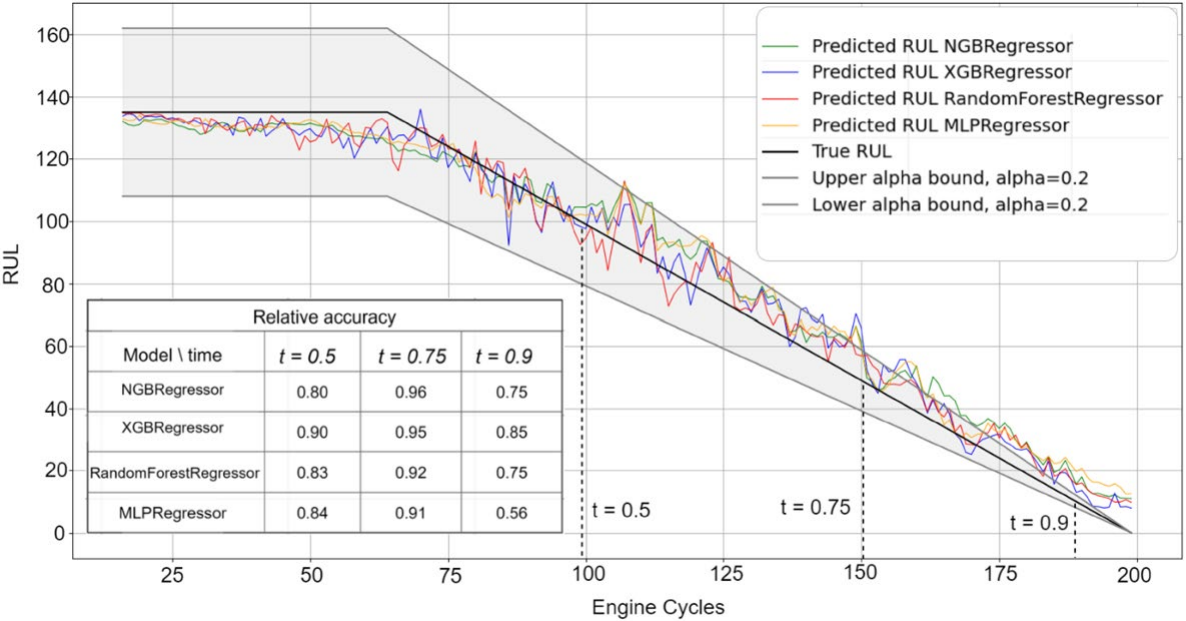
Predicted vs true RUL for engine #54 dataset FD002.

**Figure 5:**
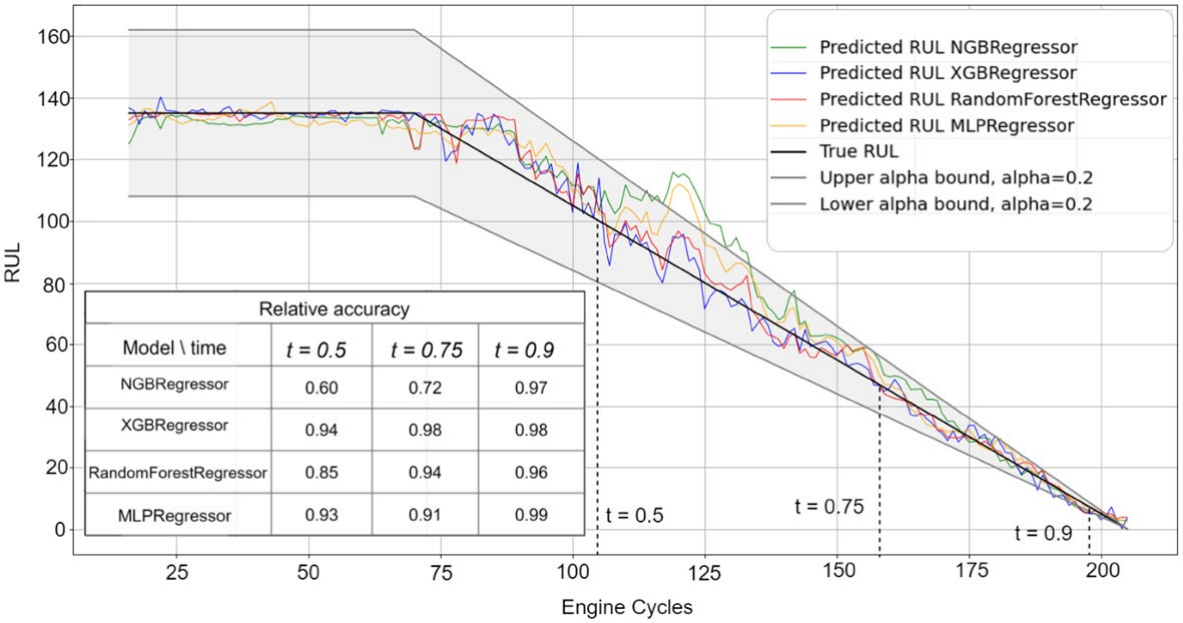
Predicted vs true RUL for engine #45 dataset FD003.

**Figure 6:**
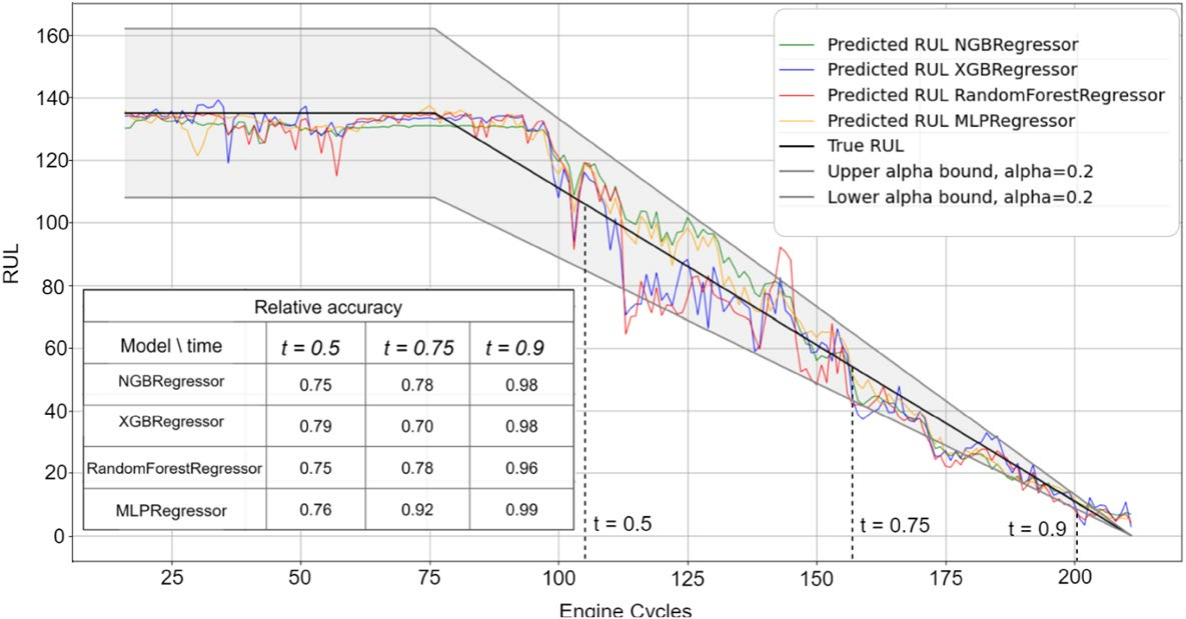
Predicted vs true RUL for engine #22 dataset FD004.

**Figure 7:**
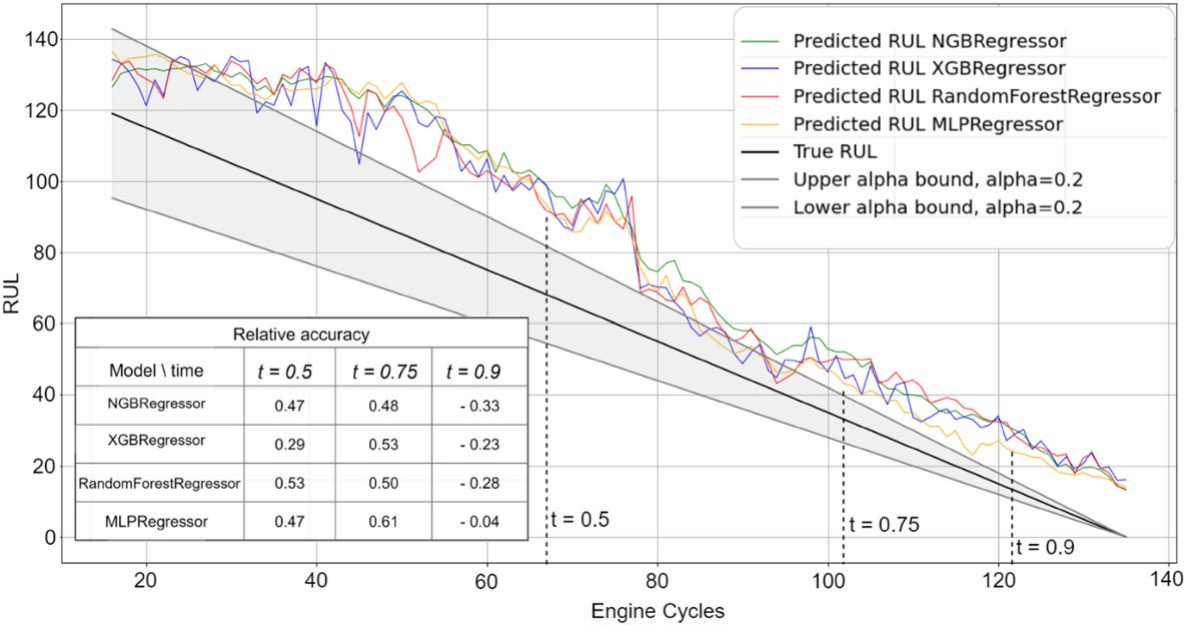
Inaccurate predicted vs true RUL for engine #91 dataset FD001.

Figures 3, 4, 5, 6 and 7 are the visual representation of the predicted RUL against the true RUL values, along with the corresponding prediction intervals: *alpha* = 0.2, *upper_bound* = *RUL* × (1 + *alpha), lower_bound* = *RUL* × (1 − *alpha*). These predictions provide a clear and concise comparison of the performance of each model, allowing us to observe the accuracy and reliability of the predictions in relation to the true RUL. By examining the prediction intervals, the level of uncertainty associated with the RUL estimates can be assessed, which is a critical aspect for making informed decisions regarding maintenance planning and resource allocation.

These figures demonstrate the effectiveness of the selected features and modeling approach in capturing the degradation patterns of the equipment over time. The close alignment between the predicted RUL and true RUL values across the different models and engines indicates a high degree of consistency in the predictive capabilities of the machine learning techniques employed in this study. However, Fig. 7 displays a prediction with most values outside the alpha bound, which could be attributed to various factors such as noise in the sensor data. Despite this, the overall degradation pattern can still be discerned alongside the true RUL.

Additionally, relative accuracy, a measure reflecting the proximity of predicted values to their true counterparts, was assessed at three distinct time points (t = 0.5, t = 0.75, and t = 0.9) across the sub-datasets FD001, FD002, FD003, and FD004. The study’s results, presented in Figs. 3, 4, 5, 6 and 7, consistently demonstrate strong model performance, validated by the high relative accuracy values obtained from randomly chosen engine in each dataset. This consistent performance across various time stages and datasets not only underscores the effectiveness of the feature selection and modeling approach in capturing time-dependent degradation patterns but also affirms its robustness and adaptability.

To evaluate the prediction, the within-interval percentages for each of the four models were calculated by determining the proportion of predictions falling within the defined interval *alpha*. These metrics provide a quantitative assessment of how accurately each model's predictions align with the true RUL and how frequently they are contained within the prediction interval. By comparing these values across the ML models, the most effective model for the given dataset can be identified.

Table 10 presents the average percentage of predictions that fall within the alpha bound for the different datasets, derived from a test sample comprising 10% of the engines from the training data. The percentages for both GA and AFICv feature selection methods hover between 70 and 80%. This demonstrates that AFICv can achieve comparable prediction accuracy to GA, but with fewer selected features. These results offer valuable insight into the accuracy and reliability of the predictive models for each sub-dataset.

**Table 10:** Average of within-interval percentage.

Model/dataset	FD001 (10 engines)		FD002 (26 engines)		FD003 (10 engines)		FD004 (25 engines)	
	GA (%)	AFICv (%)	GA (%)	AFICv (%)	GA (%)	AFICv (%)	GA (%)	AFICv (%)
NGBRegressor	77	**78**	72	**71**	80	**79**	70	**71**
RandomForestRegressor	77	**78**	71	**70**	78	**79**	70	**71**
XGBRegressor	79	**79**	70	**70**	77	**80**	72	**70**
MLPRegressor	81	**80**	72	**73**	80	**81**	70	**72**

Significant values are given in bold.

When comparing the performance of the four machine learning models, a remarkable similarity is observed in their within-interval percentages across each dataset, indicating equivalent predictive capabilities. The fluctuation in the within-interval percentages across different datasets may be attributable to the intricacies related to various operating conditions and fault modes, thereby underscoring the adaptability of the models to a multitude of scenarios.

It's noteworthy that Engine selection significantly influences the average percentage values, as demonstrated by the data in Table 11 for the FD001 sub-dataset using AFICv and GA on ten engines chosen randomly. A significant variation is observed: engine number 91 registers a meager percentage of about 1% with both methods, contrasting sharply with engine 34, which achieves up to 97% with AFICv (using only four features) and 93% with GA (utilizing 12 features). This discrepancy underscores the efficiency of AFICv.

**Table 11:** Within-interval percentage for 10 engines from FD001.

Engine	34 (%)	81 (%)	71 (%)	54 (%)	15 (%)	5 (%)	19 (%)	22 (%)	32 (%)	91 (%)
MLPRegressor (AFICv)	**97**	92	85	84	77	74	64	60	55	**0.9**
MLPRegressor (GA)	**93**	87	85	84	81	80	60	56	60	**1**

Significant values are given in bold.

### Principal component loading interpretability

Following the application of Principal Component Analysis (PCA) on our dataset, we turned our attention to interpreting the resultant principal components (PCs), the results of which can be found in Supplementary Appendix 2. Our objective was to elucidate the underlying patterns and associations captured by these components within the complex multidimensional space of our data. Specifically, we zeroed in on the top five variables with the highest absolute loadings for each component, which enabled us to highlight the most significant contributors to each pattern. Through this lens, we translated intricate statistical relationships into tangible real-world implications in the context of aircraft engine operations, thus demystifying the mechanics behind the dominant features.

- *PC1* This component is associated with root mean square, mean, and FFT coefficients of sensors measuring Total temperature at LPT outlet (T50) and Static pressure at HPC outlet (Ps30). In simple terms, PC1 is influenced by the variation, average, and frequency-related information in these temperature and pressure readings. For instance, large variations in the temperature at the LPT outlet or static pressure at the HPC outlet, as indicated by a high root mean square, might suggest unstable operating conditions^52^. Similarly, changes in the FFT coefficients could indicate periodic fluctuations in these measures, perhaps related to specific engine operating cycles or anomalies^53^.
- *PC2* primarily represents the permutation entropy from the readings of multiple sensors that measure speed and pressure (specifically sensors Nf, Nc, phi, NRc, and BPR). Permutation entropy is a statistical measure of complexity or unpredictability in a time series. Therefore, PC2 seems to capture a unique dimension of the data associated with the unpredictability of these sensor readings. In the context of an aircraft engine, the permutation entropy of speed and pressure readings may reflect engine stability or efficiency^54,55^. For instance, a highly unpredictable core speed or a drastic fluctuation in the bypass ratio might signal potential instability or inefficiency in the engine’s functioning. Conversely, a steady and predictable pattern could suggest optimal performance. Therefore, PC2 offers an important perspective on engine behavior, specifically concerning the variability and predictability of key speed and pressure parameters.
- *PC3* This component is heavily weighted by basic statistical metrics (mean, max, min) and the linear trend of the Corrected core speed (NRc). The linear trend could suggest changes in the core speed over time. For example, if we see a positive slope, it might suggest an increase in the corrected core speed over time, which could be indicative of changes in engine operation or efficiency^56^. The mean, maximum, and minimum can provide insights into the general behavior of the core speed during the operation^57^.
- *PC4 and PC5* These principal components significantly represent the variation and complexity (as indicated by permutation entropy and complexity index) in the total pressure in the bypass-duct (P15). Permutation entropy has been successfully employed in fault diagnosis in rotating machinery, as it quantitatively analyzes signals at different scales, capturing the system’s complexity^58^. A high standard deviation or permutation entropy in this sensor’s reading might suggest substantial fluctuations in the bypass-duct pressure. These fluctuations could be associated with various factors such as changes in environmental conditions, engine performance, or efficiency. Consequently, PC4 and PC5 might be capturing the overall variability and unpredictability of these pressure readings, which could be crucial for predicting the stability or efficiency of engine operations.
- *PC6 and PC7* These components are significantly influenced by the mean, trend, and autocorrelation of the total pressure in the bypass-duct (P15). Autocorrelation, a technique useful for detecting patterns or trends over time in time-series data^59^, reveals how the pressure reading at a particular time point relates to its past values. This could indicate predictable patterns or trends in the bypass-duct pressure. The effectiveness of autocorrelation in capturing long-term dependencies in the data and aiding in anomaly detection is well-documented in Ref.^60^. These patterns might be normal, possibly due to known engine cycles, or they might signal potential issues, such as periodic anomalies related to pressure fluctuations. Hence, PC6 and PC7, by capturing these long-term dependencies in the pressure data, could be significant in forecasting the health of the engine or predicting potential issues.
- *PC8, PC9, PC10, PC11, PC14, PC15* These components are dominated by the autocorrelation and partial autocorrelation of various sensors (T24, T30, P30, Nc, Ps30, NRf, NRc, BPR, W31, W32). These features measure how a sensor reading at one point in time is related to its previous readings. In the context of an aircraft engine, significant autocorrelation could indicate regular, repeating patterns in these readings, which could be normal (like due to regular cycles of the engine's operation) or problematic (like due to a recurring fault or anomaly)^61,62^.
- *PC12 and PC13* These components are influenced by the autocorrelation of the HPT and LPT coolant bleed (Sensors W31 and W32) and the standard deviation and complexity of the Bleed Enthalpy (htBleed). These might represent variability and complexity in the coolant bleed rates and the energy lost to the bleed system. Unusually high variability or complexity in these measures might indicate issues with the engine’s cooling or bleed systems, particularly in the context of the design and operation of gas turbines^63^.

## Conclusion

In conclusion, this study introduces a robust and comprehensive methodology for predicting the RUL of aircraft engines. The approach integrates advanced feature engineering, various feature selection methods, and machine learning models. The exploration of multiple feature selection techniques serves a dual purpose: to validate the efficiency of the feature engineering and dimensionality reduction process, and to benchmark the novel AFICv technique against established methods, such as GA, RFE, LASSO Regression, and FIRF.

The feature engineering phase employs a rolling time series window to extract statistically significant features from sensor data. Subsequently, the performance of four machine learning models, namely NGBRegressor, RandomForestRegressor, XGBRegressor, and MLPRegressor, is evaluated based on the selected features. Comprehensiveness in performance evaluation is ensured by considering within-interval percentages and relative accuracy metrics.

The proposed method’s effectiveness is demonstrated by its competitive performance across the C-MAPSS sub-datasets, with RMSE values of 11.8, 23, 14.6, 22.3 for FD001, FD002, FD003, and FD004 respectively. In particular, the AFICv technique exhibits efficiency by significantly reducing the number of selected features, while still maintaining comparable performance with a minimized subset of the most critical features. These results affirm the efficacy of our feature engineering and dimensionality reduction process, highlighting the potential of AFICv for future RUL prediction tasks.

While enhancing this comprehensive methodology, an interpretability perspective has been considered to bridge the gap between domain experts and the derived models. By providing a real-world context for the chosen features, we aim to facilitate a clearer understanding of their importance and relevance in the context of aircraft engines.

While the proposed methodology achieves significant results, it is important to acknowledge its limitations. The method relies on an ideal condition where complete and well-labeled data are available, which might not always be the case in real-world scenarios. Additionally, the complexity of the machine learning models used could potentially hinder real-time application due to computational costs. Furthermore, while dimensionality reduction techniques such as PCA aid in computational efficiency, they can sometimes decrease model interpretability. Efforts have been made to address this through our added interpretability section, but this remains a challenge for all model-based prognostics.

### Supplementary Information

Supplementary Information.

## Data Availability

The datasets used and/or analyzed during the current study available from the corresponding author on reasonable request.
