# Author Correction: Advancing aircraft engine RUL predictions: an interpretable integrated approach of feature engineering and aggregated feature importance

**DOI:** 10.1038/s41598-024-66604-x

**Published:** 2024-07-08

**Authors:** Yazan Alomari, Mátyás Andó, Marcia L. Baptista

**Affiliations:** 1 Faculty of Informatics, Institute of Computer Science, ELTE Eötvös Loránd University, Budapest, Hungary; 2 Faculty of Aerospace Engineering, Delft University of Technology, Delft, The Netherlands

Correction to: *Scientific Reports* 10.1038/s41598-023-40315-1, published online 18 August 2023

The original version of this Article contained an error in Figure 1, where “FD004” was omitted from the “Testing” block. The original Figure 1 and accompanying legend appear below.

**Figure 1:**
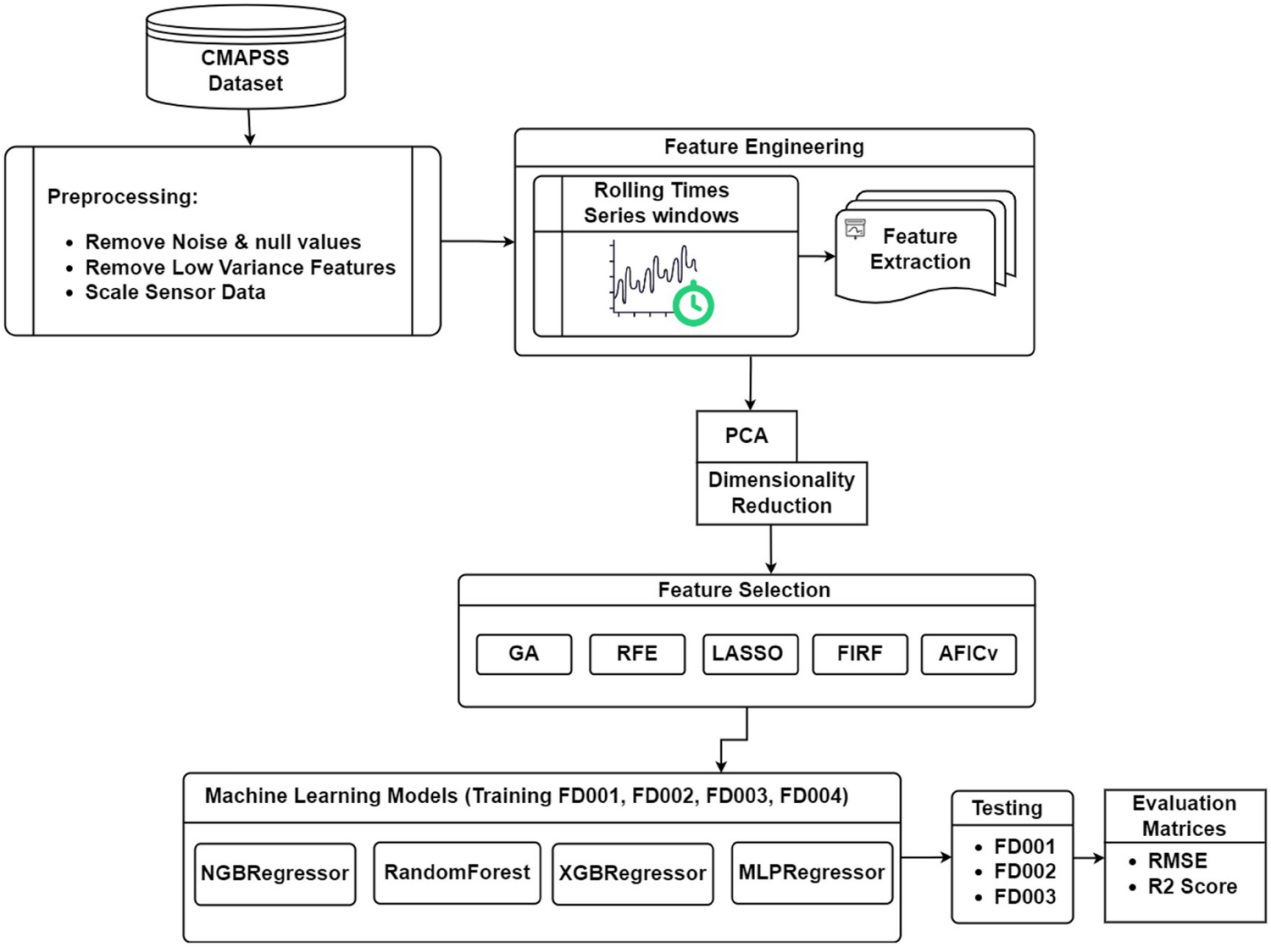
Flowchart illustrating the proposed workflow.

The original Article has been corrected.

