# Supplementary material for: Advancing aircraft engine RUL predictions: an interpretable integrated approach of feature engineering and aggregated feature importance
Source: Sci Rep. 2023 Aug 18;13:13466. doi: 10.1038/s41598-023-40315-1 (PMC10439180; doi:10.1038/s41598-023-40315-1)
Supplement: Supplementary file 1 — Supplementary Information. [file 41598_2023_40315_MOESM1_ESM.docx]

Appendix 1.

3.4.1 Genetic Algorithm-based Feature Selection

The GA searches for the best subset of features (or principal components in this case) for RUL prediction by optimizing a fitness function based on the performance of a machine learning model. The fitness function is defined as the mean cross-validated score (e.g., negative mean squared error) of the model trained on a subset of features.

The GA operates on a population of binary strings, where each string represents a candidate feature subset. The length of the string is equal to the total number of features, and each bit in the string indicates whether a particular feature is included (1) or excluded (0) from the subset [64].

The GA optimization process [65], several generations of candidate solutions are evolved using genetic operators such as selection, crossover, and mutation. The selection operator favors candidate solutions with better fitness scores, while crossover and mutation operators introduce diversity and explore new regions in the search space.

The GA optimization process proceeds as follows:

1. Initialize the population of size (50) with random binary strings representing candidate feature subsets.
2. Evaluate the fitness of each individual in the population based on the performance of the machine learning model trained on the corresponding feature subset.
3. Select the top-performing individuals using a selection strategy such as tournament selection or roulette wheel selection.
4. Create offspring by applying crossover and mutation operators to the selected individuals (*crossover probability=0.5, mutation probability=0.2*).
5. Replace the least fit individuals in the population with the newly created offspring.
6. Repeat steps 2 to 5 for a predetermined number of generations or until a stopping criterion is met.

At the end of the optimization process, the GA returns the best feature subset (i.e., the one with the highest fitness score) for RUL prediction. This final set of selected features, or principal components in our case, is then used to train the final predictive model.

3.4.2 Recursive Feature Elimination (RFE) method

This technique systematically identified the most important features in the dataset by iteratively fitting the model and removing the least significant features at each step. By employing RFE, we were able to narrow down the feature space to a smaller, yet more meaningful set, which contributed to a better understanding of the underlying relationships in the data. As a result, our model achieved higher accuracy and generalizability while also reducing the risk of overfitting. This demonstrated the effectiveness of RFE as a valuable feature selection technique for enhancing the predictive capabilities of our model [66].

3.4.3 LASSO Regularization

In our model, the LASSO (Least Absolute Shrinkage and Selection Operator) Regularization feature selection method was employed to identify the most significant features contributing to the prediction of Remaining Useful Life (RUL). LASSO is a linear regression method that utilizes L1 regularization, effectively shrinking the coefficients of less important features to zero, thereby eliminating them from the model [67]. By adjusting the regularization parameter, we were able to control the sparsity of the model and select the optimal subset of features. This resulted in a more interpretable and efficient model with reduced complexity and improved generalization capabilities, ultimately enhancing the RUL prediction performance.

3.4.4 Feature importance’s from a Random Forest

In our study, we employed feature importance’s derived from a random forest model as an effective feature selection technique. Random forests are a well-established ensemble learning method, consisting of multiple decision trees, which provide a robust and interpretable measure of feature importance by considering the contribution of each feature towards the improvement in node purity [68]. By ranking the features based on their importance scores, we were able to identify the most relevant and informative features for predicting the remaining useful life of the equipment. This approach not only improved the performance of our model but also helped reduce overfitting and computational complexity.

3.4.5 Aggregated feature importance’s with cross validation

In the proposed model, a robust feature selection technique to identify the most relevant features for various machine learning models was employed. Which efficiently captured the intricate relationships between the features and the target variable, while also reducing the complexity of the ML models. By training each model with cross-validation as shown in Figure 8, it ensured that the feature importance’s derived from the models were reliable and robust [69,70].


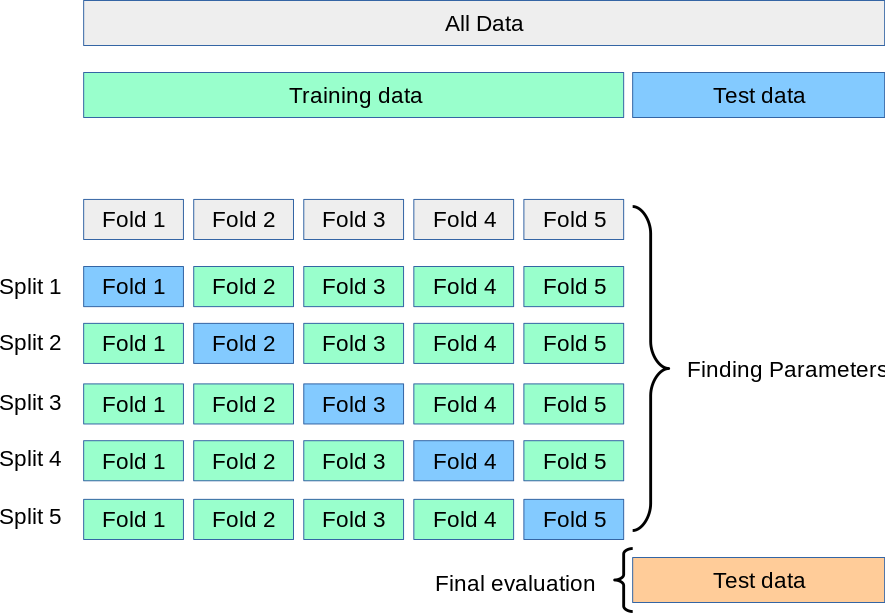


Figure 8 Cross-Validation illustration [70]

Appendix 2.

| **PC1** | Loading Values | **PC2** | Loading Values |
| --- | --- | --- | --- |
| (Ps30) Root Mean Square | 0.0822 | (NRc) Permutation Entropy | 0.1035 |
| (Ps30) FFT Coefficient | 0.0819 | (phi) Permutation Entropy | 0.1032 |
| (T50) Root Mean Square | 0.0818 | (BPR) Permutation Entropy | 0.1032 |
| (Ps30) Mean | 0.0813 | (Nf) Permutation Entropy | 0.1032 |
| (T50) FFT Coefficient | 0.0813 | (Nc) Permutation Entropy | 0.1031 |

| **PC3** | Loading Values | **PC4** | Loading Values |
| --- | --- | --- | --- |
| (NRc) Mean | 0.1538 | (P15) Standard Deviation | 0.2172 |
| (NRc) Maximum | 0.1536 | (P15) CID CE Normalize False | 0.2170 |
| (NRc) Minimum | 0.1510 | (P15) Permutation Entropy D3 | 0.2129 |
| (NRc) Linear Trend "intercept" | 0.1492 | (P15) Permutation Entropy D4 | 0.2126 |
| (NRc) Linear Trend "slope" | 0.1492 | (P15) Permutation Entropy D5 | 0.2118 |

| **PC5** | Loading Values | **PC6** | Loading Values |
| --- | --- | --- | --- |
| (P15) Root Mean Square | 0.1782 | (P15) C3 lag 1 | 0.1765 |
| (P15) Standard Deviation | 0.1485 | (P15) C3 lag 2 | 0.1746 |
| (P15) CID CE Normalize False | 0.1456 | (P15) Mean | 0.1746 |
| (P15) Permutation Entropy D3 | 0.1430 | (P15) C3 lag 3 | 0.1712 |
| (P15) CID CE Normalize True | 0.1421 | (P15) Linear Trend "intercept" | 0.1635 |

| **PC7** | Loading Values | **PC8** | Loading Values |
| --- | --- | --- | --- |
| (P15) Mean | 0.2833 | (Nc) Partial Autocorrelation lag 1 | 0.1316 |
| (P15) C3 lag 1 | 0.2688 | (Nc) Autocorrelation lag 1 | 0.1316 |
| (P15) Linear Trend "intercept" | 0.2684 | (NRf) Partial Autocorrelation lag 1 | 0.1310 |
| (P15) C3 lag 3 | 0.2657 | (NRf) Autocorrelation lag 1 | 0.1310 |
| (P15) C3 lag 2 | 0.2616 | (NRc) Autocorrelation lag 1 | 0.1302 |

| **PC9** | Loading Values | **PC10** | Loading Values |
| --- | --- | --- | --- |
| (T24) Partial Autocorrelation lag 1 | 0.2418 | (BPR) Autocorrelation lag 1 | 0.2314 |
| (T24) Autocorrelation lag 1 | 0.2418 | (BPR) Partial Autocorrelation lag 1 | 0.2314 |
| (T30) Partial Autocorrelation lag 1 | 0.2182 | (Ps30) Partial Autocorrelation lag 1 | 0.2111 |
| (T30) Autocorrelation lag 1 | 0.2182 | (Ps30) Autocorrelation lag 1 | 0.2111 |
| (T24) CID CE Normalize True | 0.2084 | (BPR) CID CE Normalize True | 0.1987 |

| **PC11** | Loading Values | **PC12** | Loading Values |
| --- | --- | --- | --- |
| (phi) Autocorrelation lag 1 | 0.2075 | (W31) Autocorrelation lag 1 | 0.2341 |
| (phi) Partial Autocorrelation lag 1 | 0.2075 | (W31) Partial Autocorrelation lag 1 | 0.2341 |
| (phi) CID CE Normalize True | 0.1826 | (htBleed) Standard Deviation | 0.2226 |
| (T24) Partial Autocorrelation lag 1 | 0.1804 | (W31) CID CE Normalize True | 0.2023 |
| (T24) Autocorrelation lag 1 | 0.1804 | (htBleed) Lempel Ziv Complexity bins 10 | 0.1885 |

| **PC13** | Loading Values | **PC14** | Loading Values |
| --- | --- | --- | --- |
| (W32) Autocorrelation lag 1 | 0.2068 | (P30) Autocorrelation lag 1 | 0.2549 |
| (W32) Partial Autocorrelation lag 1 | 0.2068 | (P30) Partial Autocorrelation lag 1 | 0.2549 |
| (W31) Partial Autocorrelation lag 1 | 0.1958 | (P30) CID CE Normalize True | 0.2227 |
| (W31) Autocorrelation lag 1 | 0.1958 | (T30) Autocorrelation lag 1 | 0.2126 |
| (htBleed) Standard Deviation | 0.1852 | (T30) Partial Autocorrelation lag 1 | 0.2126 |

| **PC15** | Loading Values |
| --- | --- |
| (T24) Autocorrelation lag 1 | 0.2365 |
| (T24) Partial Autocorrelation lag 1 | 0.2365 |
| (W32) Partial Autocorrelation lag 1 | 0.2245 |
| (W32) Autocorrelation lag 1 | 0.2245 |
| (T24) CID CE Normalize True | 0.2051 |

References

[64] Mirjalili S. Genetic algorithm. Studies in Computational Intelligence 2019;780:43–55. https://doi.org/10.1007/978-3-319-93025-1_4/COVER.

[65] Weile DS, Michielssen E. genetic algorithm optimization applied to electromagnetics: A review. IEEE Trans Antennas Propag 1997;45:343–53. https://doi.org/10.1109/8.558650.

[66] Guyon I, Weston J, Barnhill S, Vapnik V. Gene selection for cancer classification using support vector machines. Mach Learn 2002;46:389–422. https://doi.org/10.1023/A:1012487302797/METRICS.

[67] Tibshiranit R. Regression Shrinkage and Selection Via the Lasso. J R Stat Soc Series B Stat Methodol 1996;58:267–88. https://doi.org/10.1111/J.2517-6161.1996.TB02080.X.

[68] Breiman L. Random forests. Mach Learn 2001;45:5–32. https://doi.org/10.1023/A:1010933404324/METRICS.

[69] Berrar D. Cross-Validation Call for Papers for Machine Learning journal: Machine Learning for Soccer View project Cross-validation n.d. https://doi.org/10.1016/B978-0-12-809633-8.20349-X.

[70] Pedregosa FABIANPEDREGOSA F, Michel V, Grisel OLIVIERGRISEL O, Blondel M, Prettenhofer P, Weiss R, et al. Scikit-learn: Machine Learning in Python Gaël Varoquaux Bertrand Thirion Vincent Dubourg Alexandre Passos PEDREGOSA, VAROQUAUX, GRAMFORT ET AL. Matthieu Perrot. Journal of Machine Learning Research 2011;12:2825–30.
